# Animal models of antiphospholipid syndrome

**DOI:** 10.3389/fimmu.2026.1743498

**Published:** 2026-01-23

**Authors:** Chunyao Ren, Hongbin Li, Tingting Ren

**Affiliations:** 1 Department of Rheumatology and Immunology, The Affiliated Hospital of Inner Mongolia Medical University, Hohhot, China; 2 Inner Mongolia Autonomous Region Key Laboratory of Pathogenesis and Immunodiagnosis of Rheumatic and Immune Diseases, Key Laboratory of Autoimmune and Rheumatic Diseases of Inner Mongolia Medical University, Hohhot, China

**Keywords:** animal models, antiphospholipid antibodies, antiphospholipid syndrome, pathological pregnancy, thrombosis

## Abstract

Antiphospholipid syndrome (APS) is an autoimmune disorder defined by persistent antiphospholipid antibodies (aPL), thrombosis, and/or pathological pregnancy. Its phenotypic spectrum is heterogeneous and its pathogenesis remains incompletely understood. The incidence of APS increases year by year. Due to the constraints on human studies, animal models have become indispensable tools for dissecting the mechanisms of APS. The animal models accelerate the drug discovery and refine the therapeutic strategies in APS. Over the past decades, substantial methodological and translational advances have been achieved in APS animal models. In this review, we systematically summarize the current construction paradigms in thrombotic and obstetric APS animal models and highlight their respective advantages and limitations.

## Introduction

Antiphospholipid syndrome (APS) is a non-organ-specific autoimmune disease caused by the binding of circulating antiphospholipid antibodies (aPL) to phospholipids or phospholipid-binding proteins on the surface of self-cells, leading to thrombosis, pathological pregnancy, or other inflammation (1). APS can be divided into primary APS and secondary APS. The primary APS accounts for 35-50% of all cases and secondary APS accounts for 50-65% of all cases. Secondary APS is commonly seen in systemic lupus erythematosus (SLE), Sjogren’s syndrome (SS), and other connective tissue diseases, among which secondary APS to SLE is the most common type of secondary APS. About 20%-30% of SLE patients have medium-high titer aPL in their serum (2).

The manifestations of APS are complex and varied. Vascular thrombosis is the prominent manifestation of APS, which can occur repeatedly in multiple locations, arteries, veins, and microvessels. In APS, the most common site of venous thrombosis is the deep vein of the lower limb. The venous thrombosis can be seen in the kidney, liver, subclavian vein, retina, superior vena cava, inferior vena cava, and intracranial venous sinus. The most common site of arterial embolism is the intracranial blood vessel. The arterial embolism may also affect the coronary artery, renal artery, and mesenteric artery (3, 4).Obstetric antiphospholipid syndrome (OAPS) is a complex autoimmune disorder that significantly compromises pregnancy, manifesting as recurrent miscarriage, stillbirth, placental insufficiency, and preeclampsia (5). In a study among pregnant women with APS-related complications, the most common complication in the mother was pre-eclampsia (9.5%). Early fetal loss occurred in approximately 35% of pregnancies and late fetal loss in 17% of pregnancies. Approximately 11% of live births were premature. There is also an increased risk of maternal venous thromboembolism in APS (6).

In recent years, an increasing number of studies have defined APS as a “thrombotic inflammatory disease” (7, 8). This shift originated from the “secondary strike” hypothesis and the concept of the “immune thrombosis”. Persistent elevation of aPL creates a microenvironment prone to thrombus formation *in vivo* (the “primary strike”), while thrombus development remains sporadic. Under the “secondary strike”, such as infection, surgery, or pregnancy, excessive activation of the coagulation system triggered by activated endothelial cells (ECs) and platelets disrupts the balance between coagulation and anticoagulation, ultimately resulting in thrombosis (8, 9).

aPL is not only a marker of APS, but also a key driver of APS (10). Now the aPL used for APS diagnosis includes lupus anticoagulant (LA), anti-cardiolipin antibody (aCL), and anti-β2-glycoprotein I antibody (aβ2GPI) (11). Among all the aPL, aβ2GPI-dependent aPL plays an important role in vascular, obstetric, and catastrophic APS (12). The signaling pathway activated by aPL depends on the individual receptors engaged and the cell type (13). aPL activate ECs and monocytes, which leads to increased expression of vascular cell adhesion molecules, pro-inflammatory cytokines, and chemokines, along with the production of type I interferon. Furthermore, aPL triggers the activation of the complement system, platelets, and neutrophils, inducing the expression of tissue factor (TF), the release of Neutrophil extracellular traps (NETs), and high mobility group box 1(HMGB1)-mediated inflammation-coagulation cascades. TF serves as a critical initiator of coagulation. NETs released by activated neutrophils promote thrombosis through their structural properties. HMGB1, a key inflammatory mediator, further exacerbates the vicious cycle between coagulation and inflammation. Collectively, these processes lead to marked inflammatory responses and pathological thrombosis (14, 15).

The traditional view holds that placental thrombosis impairing maternal-fetal blood exchange serves as the primary pathogenic mechanism of obstetric antiphospholipid syndrome. However, histopathological findings indicate that thrombosis or infarction is not universally observed (16). aPL directly disrupts the anticoagulant annexin A5 shield on the surface of trophoblast cells, induces trophoblast apoptosis, triggers acute and chronic inflammatory responses in placental tissues, and leads to increased complement deposition products in the decidual basement membrane, ultimately resulting in pregnancy loss (17).

Complement activation is a central effector mechanism in both thrombotic and obstetric APS. In OAPS, classical pathway initiation is key. Passive transfer of human aPL-IgG induces substantial C4d deposition in wild-type mouse placenta, absent in C1q/factor D-deficient mice (18). Similarly, aβ2GPI administration causes embryonic resorption with placental C3b/c deposition (19). Seminal work by Fischetti et al. first showed aPL antibodies induce complement-dependent thrombosis in LPS-primed rats, an effect abolished in C6-deficient rats or by C5 blockade (20). Pierangeli et al. confirmed that aPL monoclonal antibodies fail to enhance thrombosis in C5-deficient mice, replicable in wild-type mice with anti-C5 antibody (21). Agostinis et al. demonstrated constitutive binding of β2GPI/antibody complexes to decidual endothelium and trophoblast, causing local complement deposition and pregnancy loss, while systemic vascular endothelial binding requires an inflammatory “second strike” to trigger complement-facilitated thrombosis (22). Thus, complement activation serves as a final common pathway, with its initiation and consequences shaped by vascular bed and co-factors, unifying APS pathogenesis.

**Figure 1:**
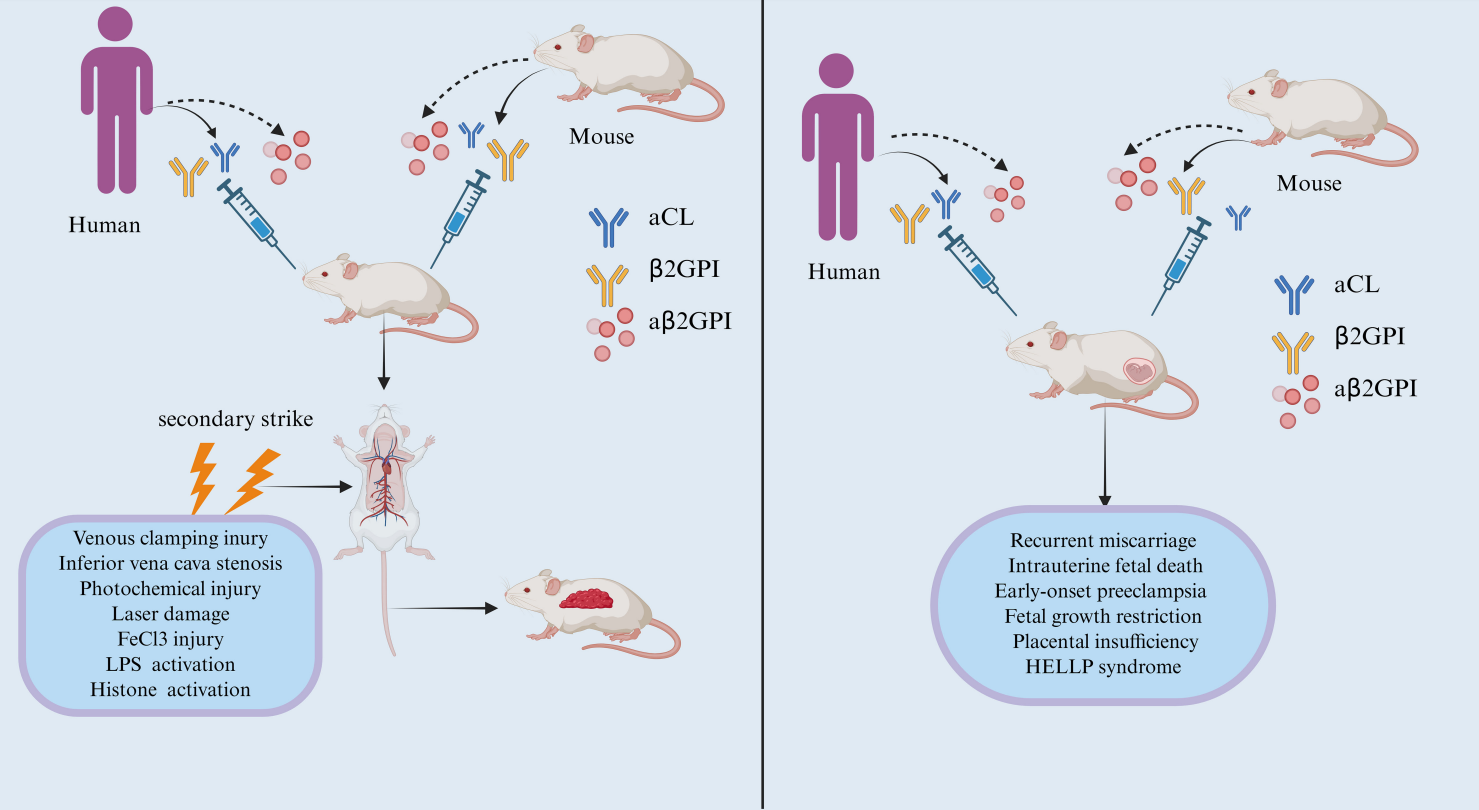
Induced thrombotic APS model and obstetric APS model. (Left) Induced thrombotic APS model: Passive immunization involves injecting antibodies such as aβ2GPI into recipient mice to establish circulating antibodies against β2GPI. Active immunization entails transferring antigens like β2GPI into recipient mice to induce the production of corresponding autoantibodies. The thrombotic APS model requires “secondary strike”. Common surgical methods for modeling include venous clamping injury, inferior vena cava stenosis, photochemical injury, laser damage, FeCl3 injury, LPS activation, Histone activation. Following “secondary strike”, thrombus formation occurs in the mice. (Right)Induced Obstetric APS model: The immunization modeling method is as described above. Once recipient mice produce antibodies such as aβ2GPI, no additional “secondary strike” is required for the mice to develop pregnancy complications, such as recurrent miscarriage, intrauterine fetal death, early-onset preeclampsia, fetal growth restriction, placental insufficiency, HELLP syndrome.

Due to ethical and practical constraints on human experimentation, animal models have become the primary means for studying disease processes and treatments (23). Currently, various animal models are available to investigate the etiology, pathogenesis, influencing factors, and therapeutic targets of APS (24). An appropriate animal model can help resolve many issues surrounding APS and provide more direct evidence for the pathogenic role of aPL. This article primarily summarizes the establishment of animal models for thrombotic APS and obstetric APS. **Figure 1** illustrates the difference in the establishment of thrombotic and obstetric antiphospholipid syndrome animal models, the thrombotic model relies on a 'secondary hit' to induce thrombosis, while the obstetric model can spontaneously produce pregnancy complications in the presence of antibodies.

## Constructing a thrombosis model of APS

### Immunological model construction

#### Passive immunization

In research on APS, passive immunization refers to the method of inducing disease phenotypes through the direct administration of specific antibodies (25). Currently, several strategies based on passive immunization have successfully established APS animal models. Pierangeli et al. (26) injected purified IgG from the serum of APS patients into CD1 mice and induced thrombosis via compression of the femoral vein. Results showed that compared to the NH-IgG-injected group, the APS-IgG group exhibited significantly increased thrombus volume and markedly elevated leukocyte adhesion to ECs. The study indicates that activation of ECs by aPL antibodies *in vivo* may create a prothrombotic state on ECs, which may be the first pathophysiological event of thrombosis in APS. J Cohen et al. (27) injected aCL-IgG/IgM derived from serum of APS patients into BALB/c mice, resulting in sustained high titers of aCL-IgG in the mice, accompanied by prolonged activated partial thromboplastin time (APTT) and thrombocytopenia. Mice injected with aCL-IgG exhibited reduced fertility, increased resorption rates in pregnant mice (equivalent to human fetal loss), and significantly higher average embryo and placental weights compared to mice injected with control IgM. M Blank et al. (28) passively transferred mouse monoclonal aCL produced by conventional hybridoma technology into mice. Researchers observed reduced fecundity rates, elevated embryo resorption indices, decreased number of embryos per litter, and reduced average embryo and placental weights compared to mice infused with corresponding control immunoglobulins. However, this approach lacks sufficient data to determine which antibody best replicates the APS disease state. However, this approach lacks sufficient data to determine which antibody best replicates the APS disease state. Another method for passively inducing APS is via bone marrow transplantation. M Blank et al. (29) induced APS in BALB/c mice by intraperitoneal injection of the aCL-H3 monoclonal antibody, establishing a donor model. They transplanted either total bone marrow cells or T-cell-depleted bone marrow cells from donor mice into naive BALB/c mice, which had received 8 Gy of whole-body irradiation. Mice receiving whole bone marrow cells subsequently developed high levels of aCL, antiphosphatidylserine antibody (aPS), antiphosphatidylinositol antibody (aPI), antiphosphatidylcholine antibody (aPC), and antiphosphatidylethanolamine (aPE) antibodies, along with thrombocytopenia, prolonged APTT, and high fetal resorption rates. In contrast, mice injected with T-cell-depleted bone marrow cells exhibited virtually none of these abnormalities, demonstrating that T cells are a critical component in the passive immunization of APS.

#### Active immunization

The active immunization is to immunize animals directly with phospholipid or its bound protein as the antigen, inducing the body to produce aPL, leading to the occurrence of APS. In APS, the most characteristic autoantigen is not phospholipids, but a lipid-binding protein --apoprotein H (APOH)-- that circulates at high levels in the blood (100-200µg/mL), and its mature protein is β2GPI protein (25, 30). *In vitro*, autoantibodies against β2GPI can activate multiple cell types, including ECs, platelets, monocytes, and neutrophils (31). Active immunization is one of the key methods for establishing animal models of APS. However, current research primarily focuses on inducing OAPS phenotypes, while exploration in thrombotic APS models remains extremely limited. This imbalance in research distribution may be related to the tissue-specific distribution of β2GPI and differences in its pathological mechanisms. Agostinis et al. (22) have provided data indicating that β2GPI injected into mice localize preferentially on the endothelium of the uterine vessels and at the implantation sites both on ECs and trophoblast in pregnant mice. In addition, the distribution pattern of this molecule is not modified by the presence of circulating antibodies that are only associated with the occurrence of an increased rate of fetal loss. Therefore, the selective localization of β2GPI in placental tissues enables antibodies binding to these sites to directly induce localized damage without requiring an additional “secondary strike”. In contrast, during systemic thrombosis, aPL typically necessitate synergistic inflammatory stimuli or other “secondary strike” to trigger significant thrombotic events (20, 32). The “secondary strike” effect of LPS was substantiated by pivotal *in vivo* evidence from Fischetti et al. (20). Their work demonstrated that while infusion of aβ2GPI- IgG alone had no procoagulant effect, it caused rapid endothelial deposition of fibrinogen followed by platelet-leukocyte aggregation and thrombotic occlusions in rats that had received a prior intraperitoneal injection of LPS. This study provided the first direct experimental proof that an inflammatory trigger is required to unlock the thrombogenic potential of aPL antibodies *in vivo*, laying the groundwork for the ‘two-hit’ hypothesis in APS thrombosis. Pierangeli et al. (33) established an animal model to investigate the thrombogenic aPL activity in lipopolysaccharide (LPS) - responsive and non-responsive (LPS-/-) mice. The results showed that IgG-APS induced significantly smaller thrombi and fewer adherent WBCs in LPS-/- mice than in LPS+/+ mice, and it triggered higher TF activity in the carotid artery homogenates of LPS+/+ mice than in LPS-/- mice. This could be indicative of the “second strike” induced by LPS. These findings confirm the strong immunogenicity and pathogenic potential of β2GPI, supporting its critical role as a core antigen in the construction of APS animal models.

### Mechanical damage modeling

The mechanical injury model is characterized by ligating or compressing target blood vessels, thereby inducing localized blood stasis, hypoxia, and endothelial denudation. This procedure induces thrombosis by exposing subendothelial collagen, which stimulates the release of fibrinogen-dependent thromboplastin and platelet adhesion/activation. Subsequent platelet aggregation activates the coagulation cascade, leading to fibrin network formation and intravascular thrombus development. This model effectively recapitulates the pathophysiology of thrombosis as seen in clinical scenarios of venous compression or physical trauma (34).

### Femoral venous clamping injury

The femoral vein clamping model was first established in CD1 mice by Pierangeli et al. in 1994 (35). Owing to the strain’s considerable genetic diversity and adaptability, this model has since become a frequently used method in thrombosis studies. To induce non-occlusive thrombus formation within the venous lumen, a standardized clamping pressure was applied to the mouse femoral vein. In this method, approximately 0.5 cm of the femoral vein was exposed. A clamp applying a pressure of 1500 g/mm² was then used to induce endothelial injury and thrombosis. Images of the thrombus-vein interface were acquired after 1 min of clamping. The thrombus area was quantified by tracing its outer boundary in the digital images, and the timing of thrombus formation and lysis was recorded (36). Willis et al. (37) employed site-specific polyethylene glycol (PEG) chemical modification to construct a larger molecular weight dimeric inhibitor (DI). They induced endogenous aβ2GPI production in C57BL/6J mice by immunized with human β2GPI, and subsequently induced thrombosis by applying compression to the femoral vein. They demonstrated that PEG-DI inhibited thrombosis formation and reduced TF expression in these mice. The venous clamp model can visualize the progression of thrombosis in real time and is an ideal choice for studying thrombus propagation and dissolution (25).

### Inferior vena cava stenosis model

The IVC stenosis model induces venous thrombosis by creating a partial blood flow restriction in the mouse inferior vena cava without causing endothelial injury, making it particularly suited for studying deep vein thrombosis in the context of APS (35). Pierangeli et al. (36) purified total IgG, IgM, and IgA from the serum of APS patients and administered these immunoglobulins to C57BL/6J mice via tail vein injection. A laparotomy was performed to expose the IVC. A segment of the IVC caudal to the left renal vein junction was then carefully dissected. A 30-G blunt needle was placed underneath this segment as a spacer, and a 7–0 Prolene suture was looped around both the IVC and the needle. Subsequent removal of the needle created a consistent, approximately 90% stenosis of the vessel lumen. The IVC was harvested for thrombus quantification 6-48h after surgery. The results showed that IgG, IgM, and IgA from APS patients could all induce venous thrombosis in C57BL/6J mice, with IgG being the most potent subtype. This model induced an approximate 80% reduction in blood flow, which triggered complete occlusive thrombosis within 24–48 hours in 60% of the mice. Meng H et al. (38) established a model by administering purified IgG from APS patients to female C57BL/6 mice via tail vein injection, followed by partial ligation of the IVC to induce venous stasis. The results demonstrated that APS-derived IgG binds to and activates neutrophils, triggering excessive NETs formation. These NETs, acting as both a scaffold and a procoagulant matrix, markedly potentiated thrombus formation under venous stasis. The thrombi generated in this stenosis-induced model exhibit structural and histological features resembling those of human thrombi, and can be reproducibly induced without endothelial denudation. However, its primary limitation lies in the significant variability in thrombus size, potentially attributable to anatomical variations in mouse vasculature (25).

### Photo-injury model

#### Photochemical injury model

The PIM involves two key steps: first, intravenous administration of the photosensitizing dye Rose Bengal; then, transillumination of the mouse carotid artery with green light from a filtered xenon lamp. Upon light activation, the dye generates ROS, which induces direct endothelial damage and subsequently triggers acute thrombosis (39). Jankowski et al. (40) first introduced this model into APS research, using hamsters to validate the prothrombotic effects of β2GPI-dependent aPL. At 55 min pre-experiment, the human aβ2GPI monoclonal antibody GPI or its F(ab’)_2_ fragment was administered via the tail vein. After right jugular vein catheterization, rose bengal was injected through the catheter, followed by a 3-min application of 540 nm green light to the left common carotid artery via a 3 mm optical fiber. Results demonstrated a significant reduction in occlusion time from 19.3 min in the control group to 7.8 min in the GPI-treated group, accompanied by a twofold increase in thrombus area. Although administration of the F(ab’)_2_ fragment also led to a shortening of occlusion time relative to controls, this effect was markedly weaker than that of the full antibody. These findings suggest that aPL exacerbates photochemically-induced thrombosis primarily through an Fc-dependent mechanism. While this model is highly standardized through defined dye dosages and excitation parameters, it is limited to replicating acute, focal endothelial injury via light irradiation. This pathogenesis is distinct from the chronic, multifactorial nature of human thrombosis; thus, the model is currently suited primarily for research on acute thrombosis (25).

#### Laser damage model

Laser injury is a relatively new method that can standardize vascular injury with automatic and predefined injury patterns, thereby precisely controlling the size and location of injury and ablation laser dose (41). Mice were placed on a fluorescence microscope to observe blood flow in small vessels within the dorsal skin fold chamber (DSC) window. A high-energy solid-state laser MiniliteI was used to irradiate small veins with a diameter of 50-80 μm at 10Hz for 30 seconds. Images were captured every 5s for 10 min (42). Passam et al. (43) employed this methodology to investigate thrombosis in both wild-type and β2GPI-deficient mice. Their findings revealed increased thrombosis in β2GPI-deficient mice, demonstrating the physiological role of β2GPI as a natural anticoagulant. The study further suggested that the V-domain of β2GPI could be utilized in antithrombotic therapies targeting APS.

The laser injury model was also used to induce testicular artery microvascular thrombosis. Following exposure of the testis and cremaster muscle on a specialized microscope tray, the tissue was continuously perfused with thermo-controlled, oxygenated (95% N2, 5% CO2) bicarbonate-buffered saline throughout imaging (44). To induce endothelial injury and monitor flow in real time, a target vessel was focused under the microscope and irradiated for 3–5 min with a 540 nm microdot laser system, while fluorescence data were captured simultaneously. Ariela Arad et al. (45) injected purified aβ2GPI-IG, aβ2GPI-negative IgG, and IgG from normal human serum into C57BL/6 mice before laser injury. Live microscopy confirmed that the presence of aβ2GPI significantly increased the thrombus area. Valerie Proulle et al. (46) demonstrated in a laser-induced thrombosis model of C57BL/6J mice that the aβ2GPI/β2GPI complexes formed *in vivo* were primarily localized on the surface of platelet thrombi rather than on vascular endothelium. This model facilitates repeated thrombus induction in the same animal, permitting real-time intravital analysis of thrombus formation and composition via a brief surgical procedure. A major limitation, however, is the gender bias intrinsic to the classic testicular artery model, which relies on male-specific anatomy and thus cannot be used to study thrombosis in females (41).

#### Chemical damage model

In the past 20 years, the FeCl3 injury model has been widely used in thrombosis research of various vascular beds such as the carotid artery, femoral vein, and mesenteric blood vessels (41). In the FeCl3-induced arterial thrombosis model, the target carotid or mesenteric artery was exposed. A piece of filter paper saturated with FeCl3 was then applied topically to the adventitial surface for 1–5 min. Following FeCl3 application, thrombus formation commenced within a 30-min window (25). Real-time intravital microscopy, combined with Doppler flowmetry and fluorescence imaging, enables the comprehensive visualization of dynamic thrombotic processes—from initial blood flow and platelet aggregation to final thrombus formation—throughout the entire experiment (47). Lin et al. (48) evaluated the impact of varying FeCl3 concentrations (30%, 40%, 50%, 60%) and exposure durations (3–10 min) on thrombus formation in the carotid arteries of Sprague-Dawley rats. Owing to the larger vessel diameter and higher blood flow velocity in rat carotid arteries, effective thrombosis induction required both higher FeCl3 concentrations and relatively prolonged exposure times compared to murine models. The results demonstrated that under identical exposure conditions, 50% FeCl3 produced more consistent and robust thrombosis than the other concentrations tested. A 10-min application of 50% FeCl3 reliably induced stable occlusive thrombi. Notably, these thrombi did not undergo spontaneous thrombolysis within 24h. In subsequent interventional experiments, administration of recombinant tissue plasminogen activator (rt-PA) resulted in dose-dependent partial thrombolysis, the efficacy of which was closely associated with the timing of rt-PA administration. In 2016, LaPlante et al. (49) used C57BL/6 mice and a TLR4-deficient strain to explore the role of TLR4 in APS-related thrombosis. Given the fine blood vessels and relatively slow blood flow in C57BL/6 mice, this study treated exposed carotid arteries with a 5% FeCl3 solution for 15 min. The results showed that wild-type mice rapidly developed thrombi after FeCl3 injury, while TLR4-deficient mice exhibited significantly reduced thrombosis. This difference indicates that aPL-mediated thrombotic effects depend on the presence and activation status of TLR4.

The femoral vein is typically selected for inducing venous thrombosis in FeCl3 injury models due to its superficial anatomical location, moderate lumen diameter, presence of physiological valve structures, and relatively slow blood flow velocity. The femoral vein is exposed and carefully dissected free from the adjacent artery. A piece of filter paper saturated with FeCl3 is then placed on its surface. After 1–5 min of contact, the paper is removed to induce thrombus formation within the venous lumen. The resulting thrombus is typically collected after 20 min for weighing and downstream analysis (50). This model produces occlusive thrombi with high reliability and reproducibility. The resultant thrombus weight exhibits a dose- and time-dependent relationship with the applied FeCl3.

The FeCl3 model is also suitable for microcirculation thrombosis studies, in which mesenteric vessels are commonly used because they are easy to observe under the microscope. To establish the model, the mesentery is exteriorized and maintained in a warm, saline-perfused bath. Following intravenous administration of Rhodamine 6G (0.3%) to label blood cells, the mouse is placed under a fluorescence microscope. Thrombosis is then induced by topical application of a 10% FeCl3-saturated filter paper (1–2 mm²) to the mesentery, with the entire process recorded in real time (51). Ramesh et al. (52) employed this model by pre-injecting eNOS+/+ or eNOS-/- mice with aPL (or NH-IgG as a control). 24 hours later, thrombosis was induced in the mesenteric microcirculation with 10% FeCl3. While aPL increased leukocyte-endothelial interactions and decreased leukocyte velocity in eNOS+/+ mice, it exerted minimal effects on these parameters in eNOS-/- mice.

The FeCl3 thrombosis model is highly sensitive to anticoagulant and antiplatelet drugs, and has become the preferred model for evaluating new thrombolytic strategies (41). Compared with carotid artery models, the mesenteric FeCl3 model offers advantages such as minimal surgical trauma, simplified procedures, and superficial blood vessels that facilitate *in vivo* microscopic imaging. However, the oxidative damage and endothelial detachment caused by this model differ from the pathogenesis of inflammation-associated thrombosis, making it unsuitable for studying thrombosis primarily driven by endothelial inflammation (53).

#### Bacterial lipopolysaccharide activation model

The mouse LPS initiation model was used to study thrombosis related to APS in the mesenteric microcirculation (54). In contrast to direct physico-chemical injury models (e.g., mechanical, photochemical, or FeCl3), the LPS-induced model recapitulates APS microvascular thrombosis by leveraging systemic inflammation. It amplifies the procoagulant effect of aPL through a thrombo-inflammatory cascade, thereby simulating the “secondary strike” phenomenon observed in the disease. In the study by Fabio Fischetti et al. (20), Wistar rats were treated with LPS intraperitoneally. At 2.5 hours post-injection, APS-IgG and the fluorescent cell label rhodamine 6G were co-administered. Upon exteriorizing the mesentery for intravital microscopy, stable platelet-leukocyte microaggregates were observed within 10–15 min. These aggregates progressively enlarged (25–30 min) and increased in number (by 2-fold at 60 min), causing partial/complete occlusion and markedly reduced flow in distal arterioles. This model confirms that LPS promotes the binding of β2GPI to vascular endothelium, leading to significantly increased leukocyte-platelet aggregation and thrombus area within mesenteric microvessels. LPS can induce consistent and repeatable microvascular thrombosis without local vascular damage, and supports high-precision microcirculation dynamic analysis. Because the LPS-priming model does not rely on a local vascular insult to induce thrombosis, this model might better mimic clinical thrombosis than models that employ chemical or physical damage (54). Raschi et al. (55) demonstrated for the first time that aβ2GPI activate endothelial cells via a MyD88-dependent signaling pathway, which is the same pathway utilized by bacterial LPS and interleukin-1 (IL-1). This finding suggests the involvement of members of the Toll-like TLR family in this process. Due to its reliance on complex laparotomy and prolonged catheterization procedures that cause significant trauma and extend surgical time—this model may be unsuitable for laboratories lacking specialized expertise in intravital microscopy and vascular surgery.

#### Histone (calf thymus histones) activation model

The CTH initiation model is characterized as a non-local inflammatory stimulation model, whereby thrombosis is induced via intravenous injection of cell-free CTH. This model is particularly useful for investigating the pathogenic role of anti-phosphatidylserine/prothrombin antibodies (aPS/PT) in APS (56). Elevated levels of aPS/PT are closely associated with increased APS severity, and CTH has been identified as a key mediator in the endothelial activation that drives inflammatory thrombosis (57). MaiYamada et al. (56) induced thrombosis in Wistar rats by sequential intravenous injection of calf thymus histone and, two hours later, an aPS/PT. Examination at 72h showed extensive microvascular thrombi in multiple organs, with the thrombus area exhibiting a linear dose-response relationship to the aPS/PT administered. This model is easy to operate and avoids complicated surgical procedures. While its non-invasive nature precludes real-time observation of thrombus dynamics, the model also poses challenges for studying specific vascular beds due to the stochastic nature of thrombosis (25). We have summarized in **Table 1** the methodologies for establishing thrombotic APS models, along with their respective advantages and disadvantages.

**Table 1:** Summary of thrombotic APS animal model construction.

Category of model	Modeling method	Applicable mice	Superiority	Shortcoming
Mechanical damage modeling	Femoral vein stasis	CD1、C57BL/6	Safe, large and stable thrombus.	Advancing against blood flow, causing widespread endothelial injury
	IVC narrow	C57BL/6	Constant blood flow, intact endothelium, structurally human-like thrombosis.	Variable thrombus size, propagation along blood flow, no real-time observation
Photo-injury Model	Photochemical damage	Guinea rat, C57BL/6	Standardized and highly repeatable	Thrombosis via rapid, uncontrollable local injury
	laser damage	C57BL/6	High automation, uniform thrombus size, accurate positioning.	Non-conformity with human thrombotic mechanism
Chemical damage model	FeCl3 chemical damage	C57BL/6, SD rats	Simple operation, real-time monitoring, rapid thrombus formation	Vascular wall damage and failure to simulate clinical thrombosis formation
LPS activation model	LPS activation model	Wistar rats, C57BL/6	Consistent thrombosis visualized in real time	Complicated, time-consuming.
Histone (Calf thymus histones, CTH) activation model	CTH activation model	Wistar rats	No surgical operation is required, and it is suitable for studying the role of aPS/PT-mediated thrombosis in APS.	Not suitable for thrombosis studies in specific areas.

#### Constructing an obstetric antiphospholipid syndrome model

Obstetric Antiphospholipid Syndrome (OAPS) is clinically defined by a spectrum of serious pregnancy complications, including recurrent miscarriage, intrauterine fetal death, early-onset preeclampsia, fetal growth restriction, placental insufficiency, and HELLP syndrome (characterized by hemolysis, elevated liver enzymes, and low platelet count) (58). Thrombotic events were initially considered the major pathological cause of pregnancy complications in OAPS (59). It is now accepted that thrombosis alone cannot explain the pregnancy morbidity observed in OAPS. aPL-mediated functional damage on placental tissue and/or the inflammatory processes involving the complement cascade are the most relevant pathogenic mechanisms contributing to the poor obstetric outcome (16, 60). Each of these mechanisms is not mutually exclusive and may act together or in different combinations at different times of pregnancy. This can explain the reason why the clinical manifestations of the OAPS span from early to late miscarriages or preeclampsia (61). The pathogenesis of OAPS involves a “multiple strike” hit”mechanism. aPL causes trophoblastic dysfunction by disrupting the anti-coagulant barrier of desminin V on trophoblast cell surfaces, increasing apoptosis, inhibiting differentiation (manifested as reduced HCG secretion), and weakening matrix metalloproteinase-mediated invasion capacity. Simultaneously, aPL induces local complement deposition, neutrophil infiltration, and release of inflammatory mediators such as tumor necrosis factor-α (TNF-α) in the placenta, creating an acute inflammatory environment. It also suppresses production of key angiogenic factors like vascular endothelial growth factor (VEGF), impeding normal development of the placental vascular network. aPL also activates neutrophils to release NETs, exacerbating inflammation and vascular injury. Furthermore, aPL induces endothelial cells and other cells to release extracellular vesicles (EVs) carrying “alarm” substances like IL-1β and mitochondrial DNA. These EVs amplify local and systemic inflammatory responses by activating TLR pathways, such as TLR7 and TLR9. These interconnected mechanisms collectively contribute to placental injury and pregnancy complications (18, 62–64).

#### Induction of the OAPS model

Two primary methodologies are employed to establish animal models of OAPS (23). The first method is to inject mouse or human monoclonal and polyclonal aPL into different strains of wild female mice via the tail vein or peritoneum. The second method employs active immunization, wherein mice are vaccinated with human or mouse-derived β2GPI antigens to induce autoimmune responses leading to pregnancy loss. This induces an immune response that leads to pregnancy loss, and after successful modeling, researchers can routinely monitor clinical and serological manifestations in mice, evaluate reproductive capacity and pregnancy outcomes post-mating, and document embryo and placental weights along with embryonic development. All models developed through different methods exhibit increased embryo absorption rates and decreased embryo/placenta weights. Additionally, some experiments revealed thrombosis, necrosis, antibody deposition, inflammatory cell infiltration, and complement activation in the placenta. The model also induces hypercoagulability of blood, placental vascular endothelial damage, thrombosis, and recurrent miscarriages.

#### OAPS passive immunization

In 1991, Piona et al. (65) established an OAPS model by passive injection of monoclonal aCL-IgG, revealing pregnancy complications such as fetal loss in ICR mice. Ikematsu et al. (66) injected pregnant BALB/c mice with human monoclonal aCL-IgG and observed elevated embryo resorption rates, reduced placental weight, and extensive fibrin deposition and thrombosis in the placental decidua. Sthoeger et al. (67) intravenously administered monoclonal aCL-IgM antibodies to early-pregnant BALB/c mice, and they showed a significant increase in the embryo resorption rate and decreased fetal weight. In contrast, aCL injection during late pregnancy did not adversely affect pregnancy outcomes, suggesting that these antibodies primarily compromise pregnancy by interfering with the process of embryo implantation. Fishman et al. (68) administered 10 µg aCL/mouse via tail vein injection on gestation day 1 to female ICR mice. The fetal resorption rate in the experimental group was 32% ± 4.2 (P < 0.0005). Following *in vivo* treatment with mrIL-3, the resorption rate was reduced to only 4% (P < 0.0005). Berman et al. (69) administered 10 mg of aPL-IgG intraperitoneally on gestational days 8 and 12 in mice. They found that aPL specifically targeted decidual tissue, resulting in rapid increases in both decidual and systemic TNF-α levels. TNF-α deficiency and TNF blockade provided protective effects for the fetus. Chen et al. (18) injected C57BL/6 and C1qfDKO mice (C1q and factor D-deficient mice) with aPL-IgG and NH-IgG, respectively. After dissection, the uterus was harvested to obtain the decidua and placenta for staining, revealing no C4 deposition in the decidua or placenta of C1qfDKO mice. In contrast, human aPL-IgG-pretreated mice exhibited substantial C4d accumulation in the placenta, confirming that aPL induces fetal loss via classical complement activation. Chen et al. (19) administered 500 µg of polyclonal anti-fibrinolytic enzyme antibody intraperitoneally on gestational days 8 and 12. Pregnant mice were euthanized on day 15, revealing significant embryonic resorption in the experimental group compared to controls, along with substantial C3b and C3c deposition in the placenta.

#### OAPS active immunization

In 1998, Blank et al. (70) administered 0.1mg/mL β2GPI via tail vein injection to BALB/c mice following mating with male mice. The results demonstrated adverse pregnancy outcomes including miscarriage and fetal growth restriction. Xiao et al. (71) performed active immunization by directly injecting β2GPI into the uterus of female BALB/c mice. After generating aβ2GPI autoantibodies, these mice were mated with males. Results showed reduced pregnancy success rates, fetal malformations, and embryonic resorption. Research by Blank and Xiao collectively demonstrates that regardless of whether antibodies are introduced passively or induced through active immunization, both pathways result in the formation of high-titer antibodies prior to embryo implantation. These antibodies persistently trigger miscarriage, fetal resorption, and malformations. The adverse obstetric outcomes in APS depend on the presence of aβ2GPI immune responses during early pregnancy. In contrast, antibodies appearing in later pregnancy do not interfere with the course of gestation. Kawaguchi et al. (72) repeatedly injected fully active mouse aβ2GPI monoclonal antibody WBCAL-1 into pregnant C57BL/6J mice with FcRγ gene deficiency, successfully inducing a fetal growth restriction model in C57BL/6J mice. They first quantified and isolated the independent pathogenic role of the aβ2GPI-FcγR pathway in placental and fetal growth. C O García et al. (73)subcutaneously immunized female PL/J mice with purified human β2GPI emulsified in Freund’s complete adjuvant. The immunized mice produced aCL, anti-DNA, and antinuclear antibodies, along with a significant reduction in fetal number, confirming the pathogenic role of aPL in the PL/J mouse model of APS.

#### Spontaneous OAPS model

Spontaneous models primarily refer to disease models caused by pathogenic antibodies produced due to abnormalities in the animal’s own immune system. MRL/lpr and (NZW×BXSB)F1 mice are commonly used to establish OAPS models. After successful modeling via antigen administration, researchers can routinely monitor clinical and serological manifestations in the treated mice. This facilitates the study of underlying disease mechanisms and the exploration of potential therapeutic approaches.

MRL/lpr mice, first reported by Murphy et al. in 1978, are widely used as a model for studying SLE and can also be applied in research on secondary APS (41). These mice carry a mutation in the Faslpr gene on chromosome 19 and spontaneously develop features associated with APS (74). Gharavi et al. (75) found that, unlike other lupus-prone strains, 2-month-old female and 3-month-old male MRL/lpr mice exhibit β2GPI-independent aCL positivity. These mice demonstrated profound reproductive deficits, with a mean litter size of only 5.3 ± 2.6—significantly lower than the 7.2 ± 2.1 observed in NIH/Swiss controls (P<0.002)—along with decreased pregnancy success, closely mirroring the obstetric manifestations of human APS. In 1990, Smith et al. (76) established the suitability of the MRL/lpr mouse model for secondary APS. Serological analyses revealed significantly elevated serum aPL levels compared to normal mice, accompanied by thrombocytopenia, cerebral thrombosis, occlusive cerebrovascular disease, and perivascular lymphocytic infiltration in the choroid plexus. A major advantage of this model is its spontaneous development of APS-related traits. However, comprehensive investigation of SLE-APS etiology and pathogenesis—particularly regarding genetic, hormonal, and environmental interactions—generally requires a combination with complementary experimental systems.

(NZW×BXSB)F1 mice are F1 hybrids derived from NZW females and BXSB males, fully expressing the BXSB-derived Yaa gene. This strain serves as a model for APS-related pathology and has been employed to evaluate therapeutic interventions such as BAFF inhibitors (77). In 1992, Hashimoto et al. (78) first demonstrated that these mice produce autoantibodies against aCL in a β2GPI-dependent manner, with antibody titers increasing with age. Subsequent studies isolated pathogenic monoclonal aCL from these mice, revealing that their production depends on B-cell and T-cell collaboration and involves antigen-driven selection of specific V gene fragments. Philip Kahn et al. (77) found that injection of adenovirus expressing BAFF-R-Ig into 8-week-old and 12-week-old mice of this strain prolonged median survival and delayed the onset of proteinuria. Despite reduced serum BAFF and B cell counts, this did not affect the production of autoantibodies such as aCL or the occurrence of thrombocytopenia, laying the foundation for BAFF inhibitor therapy research in APS. We have summarized in **Table 2** the methodologies for establishing obstetric APS models, along with their respective advantages and disadvantages.

**Table 2:** Summary of OAPS animal model construction.

Category of model	Modeling method	Mouse strains	Superiority	Shortcoming
Induction of OAPS model	Passive immunization	ICR, BALB/c, C57BL/6, 129 SvEv×C57BL/6J hybrid mouse	1.Controllable, repeatable antibody dosing. 2.Direct pathogenicity observation.	1.Transient pathology; fails to replicate chronic disease. 2.Requires repeated injections to sustain model.
	active immunization	BALB/c, PL/J, C57BL/6J	1.Natural autoantibody simulation and human disease 2.relevance. Persistent pathological manifestations.	1. Long modeling cycle. 2.Individual variation in antibody titer and pathology severity.
Spontaneous OAPS model	Genetic mutation/hybridization	MRL/lpr, (NZW×BXSB)F1	1.Spontaneous SLE-APS model for genetic/immune study. 2.Natural disease onset and progression.	1.High maintenance costs. 2.Uncontrolled disease variability.

## Conclusions

Animal models of APS play an irreplaceable role in studying disease mechanisms, diagnosis, treatment, prognosis assessment, and risk model development. Currently, the most common method for establishing thrombotic APS models involves inducing thrombus formation through mechanical injury, such as compressing specific blood vessel sites in mice. Most existing animal models only partially simulate APS clinical manifestations, lacking ideal experimental tools. OAPS-induced pregnancy loss can be modeled through induced and spontaneous approaches. The aPL-induced pregnancy loss model facilitates research on antibody-mediated adverse pregnancy outcomes. It allows investigation into areas such as the mechanisms of non-standard clinical presentations associated with aPL, the pathogenic effects of non-criterion antibodies, and the relationship between aPL and infertility or recurrent pregnancy loss, all of which merit further study. Simultaneously, animal model development should increasingly align with clinical needs to further investigate APS pathogenesis and pathophysiology, target therapies for APS patients, and ultimately minimize lifelong anticoagulant therapy requirements. Such models have already proven valuable in the preclinical development of targeted therapies. Currently, clinical trials for APS are primarily focused on biologics and cellular therapies targeting B cells and plasma cells, such as BLyS/APRIL inhibitors (e.g., belimumab, telitacicept), BTK inhibitors (e.g., zanubrutinib), and CD38 monoclonal antibodies (e.g., daratumumab). Most of these studies are currently in the open-label phase 2 trial stage, aiming to evaluate the safety and efficacy of these drugs in patients with refractory, high-risk, or thrombocytopenia-associated APS (79). Macor et al. (80) developed a new targeted thrombolytic agent consisting of nanobubbles (NB) coated with recombinant tissue plasminogen activator (rtPA) and a recombinant antibody specific for cell-bound β2GPI. Their findings suggest that targeting cell-bound β2GPI may represent an efficient and thrombus-specific thrombolytic strategy in both APS-related and APS-unrelated thrombotic conditions, showcasing a promising targeted therapeutic approach. In addition, based on a deeper understanding of the pathogenesis, other promising therapeutic targets are under exploration. These include complement C5 inhibitors (e.g., eculizumab) for managing thrombotic microangiopathy manifestations, mTOR inhibitors (e.g., sirolimus) that have shown preventive effects in APS nephropathy, and novel inhibitors targeting NETs, the interferon signaling pathway, and specific autoantibodies (e.g., aβGPI domain I antibodies). Collectively, these investigational drugs represent a shift from broad-spectrum anticoagulation toward precision strategies that target specific pathogenic pathways based on molecular subtyping (79). Animal models hold immense potential to reveal unrecognized aspects of APS pathophysiology. We anticipate animal models will remain essential components of preclinical research, laying the foundation for a new era of APS clinical trials.
